# Identification of Whole-Genome Significant Single Nucleotide Polymorphisms in Candidate Genes Associated With Serum Biochemical Traits in Chinese Holstein Cattle

**DOI:** 10.3389/fgene.2020.00163

**Published:** 2020-03-04

**Authors:** Kerong Shi, Fugui Niu, Qin Zhang, Chao Ning, Shujian Yue, Chengzhang Hu, Zhongjin Xu, Shengxuan Wang, Ranran Li, Qiuling Hou, Zhonghua Wang

**Affiliations:** College of Animal Science and Technology, Shandong Key Laboratory of Animal Bioengineering and Disease Prevention, Shandong Agricultural University, Taian, China

**Keywords:** GWAS, serum biochemical traits, cattle, Chinese Holstein, SNPs, QTL

## Abstract

A genome-wide association study (GWAS) was conducted on 23 serum biochemical traits in Chinese Holstein cattle. The experimental population consisted of 399 cattle, each genotyped by a commercial bovine 50K SNP chip, which had 49,663 SNPs. After data cleaning, 41,092 SNPs from 361 Holstein cattle were retained for GWAS. The phenotypes were measured values of serum measurements of these animals that were taken at 11 days after parturition. Two statistical models, a fixed-effect linear regression model (FLM) and a mixed-effect linear model (MLM), were used to estimate the association effects of SNPs. Genome-wide significant and suggestive thresholds were set up to be 1.22E−06 and 2.43E−06, respectively. In the Chinese Holstein population, FLM identified 81 genome-wide significant (0.05/41,092 = 1.22E−06) SNPs associated with 11 serum traits. Among these SNPs, five SNPs (BovineHD0100005950, ARS-BFGL-NGS-115158, BovineHD1500021175, BovineHD0800028900, and BTB-00442438) were also identified by the MLM to have genome-wide suggestive effects on CHE, DBIL, and LDL. Both statistical models pinpointed two SNPs that had significant effects on the Holstein population. The SNP BovineHD0800028900 (located near the gene *LOC101903458* on chromosome 8) was identified to be significantly associated with serum high- and low-density lipoprotein (HDL and LDL), whereas BovineHD1500021175 (located in 73.4Mb on chromosome 15) was an SNP significantly associated with total bilirubin and direct bilirubin (TBIL and DBIL). Further analyses are needed to identify the causal mutations affecting serum traits and to investigate the correlation of effects for loci associated with fatty liver disease in dairy cattle.

Genome-wide association study was proven to be a powerful tool for detecting genetic variants associated with economically important traits, such as production (Jung et al., 2013; Yue et al., 2017; Yan et al., 2019), reproduction (Sahana et al., 2011), and disease traits (Pant et al., 2010). This study was to identify SNPs with significant association effects on serum traits in Chinese Holstein and Jersey cattle through the use of GWAS.

The experimental population consisted of 399 Chinese Holstein dairy cows, all of which were raised on the same farm. The phenotypes were the measured values for 23 serum traits with the serum being sampled from each cow at 11 days after parturition within a month (between September and October). The serum traits were adenosine deaminase (ADA), serum albumin (ALB), alkaline phosphatase (ALP), alanine transaminase (ALT), aspartate aminotransferase (AST), β-hydroxybutyric acid (BHB), cholinesterase (CHE), creatine kinase (CK), serum creatinine (CR), direct bilirubin (DBIL), glucose (GLU), high density lipoprotein (HDL), L-lactate dehydrogenase (LDHL), low density lipoprotein (LDL), non-esterified fatty acid (NEFA), serum urea nitrogen (SUN), total bilirubin (TBIL), total cholesterol (TCHO), triglyceride (TG), total protein (TP), urea acid (UA), very low density lipoprotein (VLDL), and γ-glutamyltransferase (γ-GT). The statistical summary of these phenotypes is listed in Supplementary Table S1.

All animals were genotyped with a bovine 50K SNP chip (49,663 SNPs). SNPs from the X chromosome were counted due to the overall majority of female individuals in the study population. After the data quality control procedure (Yue et al., 2017; Yan et al., 2019), 361 animals with 41,092 SNP genotypes were finally retained for the subsequent GWAS analysis. Physical map length, the number of SNPs, and the SNP density on each chromosome, before and after the data cleaning procedure, are shown in Supplementary Table S2.

A pair-wise linkage disequilibrium (LD) analysis was conducted for the Holstein population. The results showed high genome-wide similarity of LD patterns among the cattle populations (Supplementary Figure S1). The similarity might reflect the sharing of breeding histories among the cattle. Multi-dimensional scaling (MDS) analysis of 12,380 independent SNP markers (Purcell et al., 2007; Yue et al., 2017; Yan et al., 2019) with *r^2^* < 0.2 (Wang et al., 2009), using the first and the second components, indicating that there was slight population stratification (Supplementary Figure S2). To better correct cryptic population stratification, the first MDS component was used to be the covariate in the following genome-wide association analysis (Supplementary Figure S3).

According to the previous method (Yue et al., 2017), a GWAS analysis was carried out by two statistical models, a fixed-effect linear model (FLM) and a mixed-effect linear model (MLM), implemented by the PLINK software package V1.07 (Purcell et al., 2007) and the GCTA (v1.2.4) software package (Yang et al., 2011), respectively. FLM is of the form:

y=W⁢α+x⁢β+e

where **y** is a vector of phenotypic values; **α** is a vector of fixed effects including the population mean and the first MDS component; **W** is the designed matrix for fixed effects; β is the marker effect; **x** a vector of marker genotypes; and **e** is the random errors with distribution of $N\,\left(0,I\,\sigma_{e}^{2}\right)$. Here, $\sigma_{e}^{2}$ is the residual variances. For MLM, an additive genomic relatedness matrix is included to control the type I error, which is of the form

y=W⁢α+x⁢β+Zu+e

where **Z** is the designed matrix, and **u** is the vector of random effects with the distribution of $N\,\left(0,K\,\sigma_{a}^{2}\right)$. Here, $\sigma_{a}^{2}$ is the additive genetic variances and **K** is the additive genomic relatedness matrix. The other symbols are the same as the FLM. Bonferroni corrections for the genome-wide significance and suggestive thresholds (Mapholi et al., 2016; Kerr et al., 2017) were computed to be 1.22E−06 (=0.05/41,092) and 2.43E−06 (=0.1/41,092), respectively.

A GWAS based on the FLM identified 81 SNPs with genome-wide significant (1.22E−06) association effects on 11 serum traits (Table 1) in the Holstein cattle population. A GWAS based on the MLM identified 15 SNPs as having genome-wide suggestive effects on 11 serum traits (Table 2). Among these SNPs, five SNPs (BovineHD0100005950, ARS-BFGL-NGS-115158, BovineHD1500021175, BovineHD0800028900 and BTB-00442438) were identified by both the FLM and MLM to have genome-wide suggestive effects on CHE, DBIL, and LDL.

**TABLE 1:** Genome-wide significant SNPs that were identified to be associated with serum indexes in Chinese Holstein cattle using a fixed linear model.

**Trait^1^**	**SNP-name**	**Chr**	**Position**	**Model^2^**	***P*-value**	**Nearest gene**	**Distance^3^**
AST	BovineHD2600000138	26	1234319	FLM	1.29E−08	–	–
ALP	chr5 113679525^#^	5	113679525	FLM	1.13E−07	*TCF20*	Within
	BovineHD0500032827^#^	5	113679789	FLM	1.27E−07	*TCF20*	Within
	chr5 113680107^#^	5	113680107	FLM	1.13E−07	*TCF20*	Within
	chr5 113680281^#^	5	113680281	FLM	1.25E−07	*TCF20*	Within
	chr5 113682858^#^	5	113682858	FLM	8.33E−08	*TCF20*	Within
	ARS-BFGL-NGS-33155	5	113787757	FLM	1.43E−07	*LOC104972595*	U 37356
	ARS-BFGL-NGS-5845	9	99978975	FLM	2.79E−07	*LOC100336821*	Within
	BovineHD1700011465	17	41431300	FLM	2.68E−07	*RXFP1*	Within
	BovineHD2300007148	23	25747610	FLM	5.28E−07	*LOC101903077*	U 50184
	BovineHD2800010983	28	39619766	FLM	4.66E−07	*CCSER2*	Within
	BTB-00990573	28	39950373	FLM	2.35E−08	–	–
	BovineHD2800012663	28	44105256	FLM	2.03E−07	*SLC18A3*	U 18412
TCHO	BovineHD0500019371	5	69065329	FLM	7.41E−07	*APPL2*	Within
	BovineHD2600013701	26	47604960	FLM	7.46E−08	*CLRN3*	U 30491
	Hapmap28862-BTA-149586	30	125860499	FLM	2.11E−07	*PDK3*	Within
CHE	BovineHD0100005653	1	18939484	FLM	3.44E−07	*CXADR*	Within
	BovineHD0100005950*	1	20036999	FLM	7.81E−08	–	–
	BovineHD0800022235	8	74000163	FLM	1.01E−06	–	–
	Hapmap52146-ss46526966	9	95952795	FLM	3.63E−07	*SNX9*	Within
	ARS-BFGL-NGS-115719	19	48801884	FLM	1.17E−06	*SCN4A*	Within
	BovineHD2900006235	29	21755373	FLM	6.96E−07	–	–
	ARS-BFGL-NGS-115158*	29	21828399	FLM	3.02E−07	–	–
γ-GT	BTB-01806486	26	4754531	FLM	1.31E−10	*PCDH15*	Within
TBIL	BovineHD0200031819	2	110407486	FLM	1.48E−07	*EPHA4*	D 2123
	BovineHD0700001976	7	6718398	FLM	3.72E−07	*LOC100336881*	D 13347
	BovineHD0700003730	7	14112320	FLM	4.05E−07	*LOC520104*	Within
	BovineHD0700018347	7	63443211	FLM	3.58E−07	*CDX1*	U 14171
	ARS-BFGL-NGS-41157	7	73155944	FLM	5.56E−07	*TRNAC-ACA*	U 85437
	BovineHD0700021500	7	73162347	FLM	5.56E−07	*TRNAC-ACA*	U 79034
	ARS-BFGL-BAC-20850	14	9542083	FLM	7.54E−07	*PHF20L1*	Within
	BovineHD4100011001	14	9854232	FLM	1.05E−06	*KCNQ3*	Within
	ARS-USMARC-Parent-DQ846690-no-rs	14	10171919	FLM	1.06E−07	*EFR3A*	Within
	BovineHD1400002967	14	10512600	FLM	5.33E−07	–	–
	BovineHD1400003397	14	11737590	FLM	7.54E−07	*FAM49B*	U 24208
	BovineHD1500011673	15	42127831	FLM	1.83E−07	–	–
	BovineHD1500014738	15	51303719	FLM	4.17E−07	*OR52K2*	U 12321
	BovineHD1500015826	15	54790260	FLM	1.19E−06	*CHRDL2*	Within
	BovineHD1500021175*^§^	15	73378270	FLM	2.80E−07	–	–
	BovineHD1800005102	18	16301576	FLM	1.10E−06	–	–
	BovineHD1800014694	18	49879114	FLM	1.19E−06	*MAP3K10*	Within
	BovineHD1800017510	18	60710597	FLM	5.13E−07	*LOC788928*	Within
	BovineHD2200007568	22	26040853	FLM	9.60E−07	*CHL1*	U 57003
	Hapmap50029-BTA-55899	23	24181053	FLM	1.64E−07	*PKHD1*	Within
	BovineHD2800006539	28	25438915	FLM	1.13E−07	*KIF1BP*	Within
	BovineHD2800006565	28	25578865	FLM	9.04E−07	*LOC104976190*	D 4819
	BovineHD3000030626	30	110483950	FLM	1.20E−06	*RPGR*	Within
	BovineHD3000033677	30	119764357	FLM	2.51E−07	*IL1RAPL1*	Within
	Hapmap38597-BTA-41420	30	119781376	FLM	4.50E−08	*IL1RAPL1*	Within
	Hapmap56389-rs29012404	30	141044156	FLM	1.01E−06	*TLR7*	Within
DBIL	BovineHD0400011958	4	43673293	FLM	1.18E−06	*PHTF2*	Within
	BovineHD1400003397	14	11737590	FLM	1.12E−06	*FAM49B*	U 24208
	BovineHD1500021175*^§^	15	73378270	FLM	2.18E−07	–	–
	BovineHD1800017510	18	60710597	FLM	2.91E−07	*LOC788928*	Within
	BovineHD2600013030	26	46078929	FLM	5.88E−07	*ADAM12*	Within
ALT	chr26 38656980	26	38656980	FLM	1.77E−10	*RAB11FIP2*	Within
LDHL	BovineHD0100046573	1	117801064	FLM	1.17E−06	*MED12L*	Within
HDL	BovineHD0800028900*^§^	8	97883896	FLM	4.65E−07	*LOC101903458*	D 72327
	ARS-BFGL-NGS-110774	23	29305663	FLM	5.91E−07	*LOC516273*	D 4789
LDL	BovineHD0100031530	1	111405782	FLM	1.22E−06	*LEKR1*	U 38747
	Hapmap51041-BTA-72970^#^	5	22943453	FLM	6.79E−07	*EEA1*	D 11000
	BovineHD0500034561	5	118742365	FLM	8.42E−07	*LOC104972610*	D 70630
	BovineHD0700003251	7	12515656	FLM	3.96E−07	*LOC107132604*	U 41618
	BovineHD0700027357	7	93754227	FLM	3.08E−07	*LOC104968990*	D 31838
	BovineHD0700027362	7	93771183	FLM	4.78E−07	*LOC104968990*	D 48794
	BovineHD0800016421	8	54526025	FLM	9.14E−08	*PSAT1*	Within
	ARS-BFGL-NGS-24437	8	54528592	FLM	9.14E−08	*PSAT1*	Within
	BTB-01066770	8	97834727	FLM	1.87E−08	*LOC101903458*	D 23158
	BovineHD0800028900*^§^	8	97883896	FLM	3.65E−10	*LOC101903458*	D 72327
	BovineHD0800029109	8	98540784	FLM	5.57E−08	*LOC104969466*	D 86018
	BovineHD0800029198	8	98839161	FLM	1.54E−07	*LOC101903599*	D 3281
	Hapmap38716-BTA-100681	8	98861495	FLM	3.86E−07	*KLF4*	D 13170
	ARS-BFGL-NGS-115765	9	99936460	FLM	1.06E−07	*LOC100336821*	Within
	BTB-00442438*	10	89826995	FLM	1.94E−08	*SPTLC2*	Within
	ARS-BFGL-NGS-17218	10	89905548	FLM	1.23E−07	*ALKBH1*	Within
	BTB-00442692	10	89923736	FLM	4.81E−07	*SNW1*	Within
	BovineHD1000025642	10	89947904	FLM	1.20E−07	*SNW1*	Within
	BovineHD1300011342	13	39397076	FLM	5.32E−07	*SLC24A3*	Within
	BovineHD2200004015	22	13825372	FLM	4.72E−07	*LOC104975498*	Within
	BovineHD2200004029	22	13889811	FLM	3.78E−07	*CTNNB1*	Within
	UA-IFASA-9518	27	15447004	FLM	4.84E−08	*MTNR1A*	Within

^1^AST, aspartate aminotransferase; ALP, alkaline phosphatase; TCHO, total cholesterol; CHE, cholinesterase; γ-GT, gamma-glutamyltransferase; TBIL, total bilirubin; DBIL, direct bilirubin; ALT, alanine transaminase; LDHL, lactate dehydrogenase-L; HDL, high-density lipoprotein; LDL, low-density lipoprotein. ^2^FLM, fixed linear model. ^3^The distance from the SNP locus to the gene (unit: bp); U indicates that the SNP site is located in the downstream of the gene; D indicates that the SNP site is located in the downstream of the gene; Within indicates that the SNP locus is located within the gene. *Genome-wide significant SNPs that identified to have suggestive effects on serum biochemical traits through mixed-effect linear model (MLM) in Chinese Holstein cattle. ^§^Genome-wide significant SNPs that were identified to be associated with multiple serum biochemical traits through a fixed linear model (FLM) in Chinese Holstein cattle. ^#^Genome-wide significant SNPs identified in the study was also previously identified to be associated with serum biochemical traits.

**TABLE 2:** SNPs identified to have genome-wide suggestive effects on serum biochemical traits in Holstein cattle using a mixed-effect linear model.

**Trait^1^**	**SNP-name**	**Chr**	**Position**	**Model^2^**	***P*-value**	**Nearest gene**	**Distance^3^**
NEFA	BovineHD2300011114	23	38421269	MLM	9.63E−06	–	–
AST	Hapmap24000-BTA-150203	11	71811673	MLM	2.01E−05	*BRE*	Within
TCHO	ARS-BFGL-NGS-65263	8	1.07E + 08	MLM	1.16E−05	*PAPPA*	Within
CHE	BovineHD0100005950*	1	20036999	MLM	1.85E−05	–	–
	BovineHD0200000997	2	3780881	MLM	6.33E−06	–	–
	ARS-BFGL-NGS-115158*	29	21828399	MLM	1.30E−05	–	–
DBIL	BovineHD1500021175*	15	73378270	MLM	1.73E−05	–	–
CR	ARS-BFGL-NGS-1888	23	20839913	MLM	1.74E−05	*OPN5*	D 5595
CK	chr17 71438606	17	71438606	MLM	1.39E−05	*LOC104974701*	Within
BHB	ARS-BFGL-NGS-113393	6	14179168	MLM	1.31E−05	*ZGRF1*	Within
SUN	BovineHD2900002496	29	8779426	MLM	1.21E−05	*PRSS23*	U 9262
LDL	BovineHD0400034698	4	1.18E + 08	MLM	1.39E−05	–	–
	BovineHD0800028900*	8	97883896	MLM	6.29E−06	*LOC101903458*	D 72327
	BTB-00442438*	10	89826995	MLM	2.21E−05	*SPTLC2*	Within
VLDL	ARS-BFGL-NGS-114594	5	22599252	MLM	1.76E−05	*LOC107132468*	U 31230

^1^NEFA, non-esterified fatty acid; AST, aspartate aminotransferase; TCHO, total cholesterol; CHE, cholinesterase; DBIL, direct bilirubin; CR, serum creatinine; CK, creatine kinase; BHB, β-hydroxybutyric acid; SUN, serum urea nitrogen; LDL, low-density lipoprotein; VLDL, very low-density lipoprotein. ^2^MLM, mixed-effect linear model. ^3^The distance from the SNP locus to the gene (unit: bp); U indicates that the SNP site is located in the downstream of the gene; D indicates that the SNP site is located in the downstream of the gene; Within indicates that the SNP locus is located within the gene. *SNPs identified to have genome-wide significant effects on serum biochemical traits in Chinese Holstein cattle using FLM (fixed linear model).

The SNPs identified through the MLM displayed lower overlapping than those identified through the FLM. However, the set of significant SNPs from the MLM in the study was almost a subset of SNPs from the FLM. The SNPs identified through the MLM were more conservative because the MLM took into account the additive genetic effects of each animal, and the false positive rate was expected to be lower than with the FLM. In the GWAS, the FLM with the population structure fitted as covariates may not control the type I error well, while the MLM can lead to false negatives, thus missing some potentially important discoveries (Liu et al., 2016; Supplementary Figure S3). The FLM and MLM are the most popular models in the field of GWAS (Yu et al., 2006; Purcell et al., 2007; Kang et al., 2008, 2010). On the other hand, the low overlapping genome-wide significant SNPs identified from the FLM and MLM also suggest low heritability (*h*^2^) of biochemical serum traits, which could be genetically affected by minor genes.

Interestingly, both statistical models pinpointed two SNPs (BovineHD0800028900 and BovineHD1500021175) that displayed genome-wide significant (1.22E−06) association effects on serum traits in the Holstein population. The SNP BovineHD0800028900, located at the downstream of *LOC101903458* gene on chromosome 8, was identified to be significantly associated with serum high- and low-density lipoprotein (HDL and LDL). The SNP of BovineHD1500021175 on chromosome 15 was found to have significant association effects on serum bilirubin (TBIL and DBIL). Further analyses are needed to understand the mechanism for the association effects of these SNPs on serum biochemical traits (Du et al., 2013; Hu et al., 2015).

Additionally, several candidate genes or DNA regions that we found to be significantly associated with serum biochemical traits in Holstein cattle coincided with reported association effects on other traits in the literature. For example, six SNPs at the DNA region from 113.6 to 113.7 cM of chromosome 5, closely associated with *TCF20* gene, were identified to have a significant effect on the serum ALP level (Table 1). The same DNA region was reported to have a QTL associated with blood triglyceride (TAG) levels (Wu et al., 2014). As another example, Hapmap51041-BTA-72970, located at the downstream region of *EEA1* (early endosome antigen 1), was identified to be significantly associated with serum low-density lipoprotein (LDL) level in both Holstein and Jersey cattle in the study. The same region was found to be a QTL, having an effect on abomasum displacement in German Holstein cattle (Mömke et al., 2013). *MNTR1A* (melatonin receptor 1A) was previously found associated with intramuscular fat and subcutaneous fat (Yang et al., 2015) in Qinchuan beef cattle, and it was also found to be a candidate gene of serum LDL in our study.

In summary, GWAS was conducted using two statistical models on 23 serum biochemical traits in a Chinese Holstein cattle population. Eighty-one genome-wide significant (1.22E−06) SNPs were identified to have association effects on 11 serum biochemical traits through FLM. Among these SNPs, five SNPs were also identified by the MLM to have genome-wide suggestive effects on CHE, DBIL, and LDL. There were two SNPs, BovineHD0800028900 and BovineHD1500021175, that were found to be associated with multiple serum lipoprotein levels and serum bilirubin traits, respectively. The role of these identified SNPs associated with serum biochemical traits remains to be further investigated and validated in future studies. Understand their roles may increase our understanding of the underlying molecular biology of perinatal metabolic disorder, such as fatty liver disease, in dairy cows.

## Data Availability Statement

The dataset generated in this study has been deposited into the Animal QTLdb (https://www.animalgenome.org/cgi-bin/QTLdb/BT/pubtails?PUBMED_ID=ISU0115).

## Ethics Statement

All experiments were carried out according to the Regulations for the Administration of Affairs Concerning Experimental Animals published by the Ministry of Science and Technology, China (2004) and approved by the Animal Care and Use Committee in Shandong Agricultural University, Shandong, China.

## Author Contributions

KS, QZ, and ZW conceived and designed the experiments. QH, FN, CH, ZX, SW, and RL performed the experiments. KS, CN, and SY analyzed the data. ZW, CH, SW, FN, and RL contributed the reagents, materials, and analysis tools. KS, SY, and CN wrote the manuscript.

## Conflict of Interest

The authors declare that the research was conducted in the absence of any commercial or financial relationships that could be construed as a potential conflict of interest.
