# Supplementary material for: Identification of Whole-Genome Significant Single Nucleotide Polymorphisms in Candidate Genes Associated With Serum Biochemical Traits in Chinese Holstein Cattle
Source: Front Genet. 2020 Mar 4;11:163. doi: 10.3389/fgene.2020.00163 (PMC7065260; doi:10.3389/fgene.2020.00163)
Supplement: Supplementary file 1 [file Data_Sheet_1.docx]

**Table S1** Descriptive statistics of serum biochemical traits in the studied cattle population

| **Serum index trait (unit) ^1^** | **Mean** | **Max** | **Min** | **Sample Number** |
| --- | --- | --- | --- | --- |
| ADA (U/L) | 4.09 | 8.95 | 0.068 | 395 |
| ALB (g/L) | 26.60 | 38.30 | 14.40 | 382 |
| ALP (IU/L) | 49.17 | 166.00 | 19.00 | 395 |
| ALT (U/L) | 15.13 | 99.62 | 0.98 | 395 |
| AST (IU/L) | 114.20 | 411.00 | 56.00 | 395 |
| BHB (mmol/L) | 1.28 | 3.95 | 0.54 | 395 |
| CHE (U/L) | 58.43 | 129.65 | 21.62 | 395 |
| CK (U/L) | 180.12 | 2465.44 | 30.24 | 395 |
| CR (μmol/L) | 82.42 | 174.66 | 50.37 | 382 |
| DBIL (μmol/L) | 6.96 | 13.41 | 3.75 | 395 |
| GLU (mmol/L) | 3.07 | 4.96 | 0.45 | 395 |
| HDL (mmol/L) | 1.19 | 2.35 | 0.00 | 395 |
| LDHL (U/L) | 805.89 | 1746.51 | 381.47 | 395 |
| LDL (mmol/L) | 0.073 | 0.26 | 0.010 | 395 |
| NEFA (mmol/L) | 0.82 | 4.90 | 0.087 | 395 |
| TBIL (μmol/L) | 10.50 | 40.26 | 4.87 | 395 |
| TCHO (μmol/L) | 1.99 | 4.02 | 0.080 | 395 |
| TG (μmol/L) | 0.12 | 0.25 | 0.040 | 395 |
| TP (g/L) | 61.88 | 126.70 | 34.60 | 395 |
| UA (μmol/L) | 36.93 | 73.00 | 11.00 | 395 |
| SUN (mmol/L) | 3.58 | 5.83 | 1.60 | 395 |
| VLDL (mmol/L) | 0.85 | 2.93 | 0.90 | 395 |
| γ-GT (U/L) | 24.12 | 103.38 | 10.06 | 395 |

^1^ ADA, adenosine deaminase; ALB, serum albumin; ALP, alkaline phosphatase; ALT, alanine transaminase; AST, aspartate aminotransferase; BHB, β-hydroxybutyric acid; CHE, cholinesterase; CK, creatine kinase; CR, serum creatinine; DBIL, direct bilirubin; GLU, glucose ; HDL, high density lipoprotein; LDHL, lactate dehydrogenase -L; LDL, low density lipoprotein; NEFA, nonestesterified fatty acid; TBIL, total bilirubin ; TCHO, total cholesterol; TG, triglyceride; TP, total protein; UA, urea acid; SUN, serum urea nitrogen ; VLDL, very low density lipoprotein; γ-GT, gamma-glutamyltransferase.

**Table S2** Distribution of SNP markers by chromosomes before and after quality control

| **Chromosome** | **physical length**  (Mb) | **Before QC** | |  | **After QC** | |
| --- | --- | --- | --- | --- | --- | --- |
|  |  | **SNPs Number** | **Density**(SNP/Kb) |  | **SNPs Number** | **Density**(SNP/Kb) |
| 1 | 158.3 | 2599 | 16.45 |  | 2395 | 15.13 |
| 2 | 137.1 | 2209 | 16.14 |  | 2019 | 14.73 |
| 3 | 121.4 | 2081 | 17.16 |  | 1923 | 15.83 |
| 4 | 120.8 | 1942 | 16.09 |  | 1782 | 14.74 |
| 5 | 121.2 | 2184 | 18.03 |  | 1996 | 18.02 |
| 6 | 119.5 | 2071 | 17.35 |  | 1916 | 16.01 |
| 7 | 112.6 | 1873 | 16.65 |  | 1712 | 15.19 |
| 8 | 113.4 | 1839 | 16.23 |  | 1684 | 14.85 |
| 9 | 105.7 | 1827 | 17.29 |  | 1676 | 15.86 |
| 10 | 104.3 | 1745 | 16.74 |  | 1605 | 15.39 |
| 11 | 107.3 | 1775 | 16.56 |  | 1630 | 15.18 |
| 12 | 91.2 | 1425 | 15.64 |  | 1325 | 14.53 |
| 13 | 84.2 | 1550 | 18.43 |  | 1410 | 16.75 |
| 14 | 84.6 | 1492 | 17.65 |  | 1369 | 16.19 |
| 15 | 85.3 | 1406 | 16.50 |  | 1279 | 14.99 |
| 16 | 81.7 | 1309 | 16.04 |  | 1207 | 14.77 |
| 17 | 75.2 | 1243 | 16.54 |  | 1133 | 15.07 |
| 18 | 66.0 | 1226 | 18.58 |  | 1139 | 17.25 |
| 19 | 64.1 | 1225 | 19.13 |  | 1129 | 17.61 |
| 20 | 72.0 | 1343 | 18.66 |  | 1224 | 17.01 |
| 21 | 71.6 | 1189 | 16.63 |  | 1076 | 15.03 |
| 22 | 61.4 | 1024 | 16.69 |  | 938 | 15.26 |
| 23 | 52.5 | 947 | 18.06 |  | 864 | 16.47 |
| 24 | 62.7 | 1085 | 17.32 |  | 989 | 15.76 |
| 25 | 42.9 | 753 | 17.56 |  | 676 | 15.72 |
| 26 | 51.7 | 886 | 17.16 |  | 792 | 15.33 |
| 27 | 45.4 | 730 | 16.09 |  | 667 | 14.71 |
| 28 | 46.3 | 792 | 17.13 |  | 729 | 15.75 |
| 29 | 51.5 | 834 | 16.29 |  | 752 | 14.58 |
| X | 148.8 | 2351 | 15.80 |  | 2056 | 13.81 |
