# Supplementary material for: Identification of Whole-Genome Significant Single Nucleotide Polymorphisms in Candidate Genes Associated With Serum Biochemical Traits in Chinese Holstein Cattle
Source: Front Genet. 2020 Mar 4;11:163. doi: 10.3389/fgene.2020.00163 (PMC7065260; doi:10.3389/fgene.2020.00163)
Supplement: Supplementary file 2 [file Presentation_1.PPTX]

## Slide 1
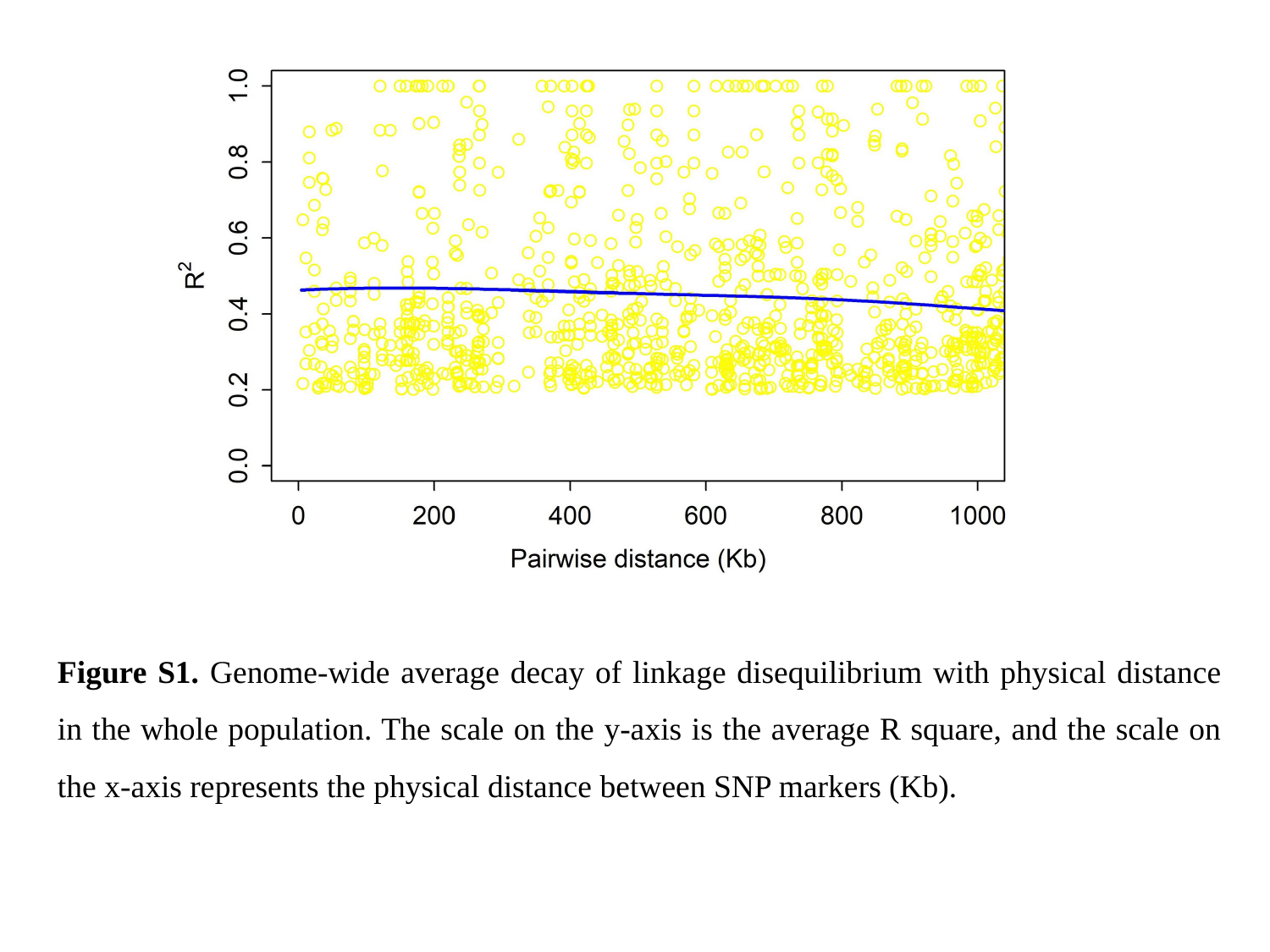

Figure S1. Genome-wide average decay of linkage disequilibrium with physical distance in the whole population. The scale on the y-axis is the average R square, and the scale on the x-axis represents the physical distance between SNP markers (Kb).

## Slide 2
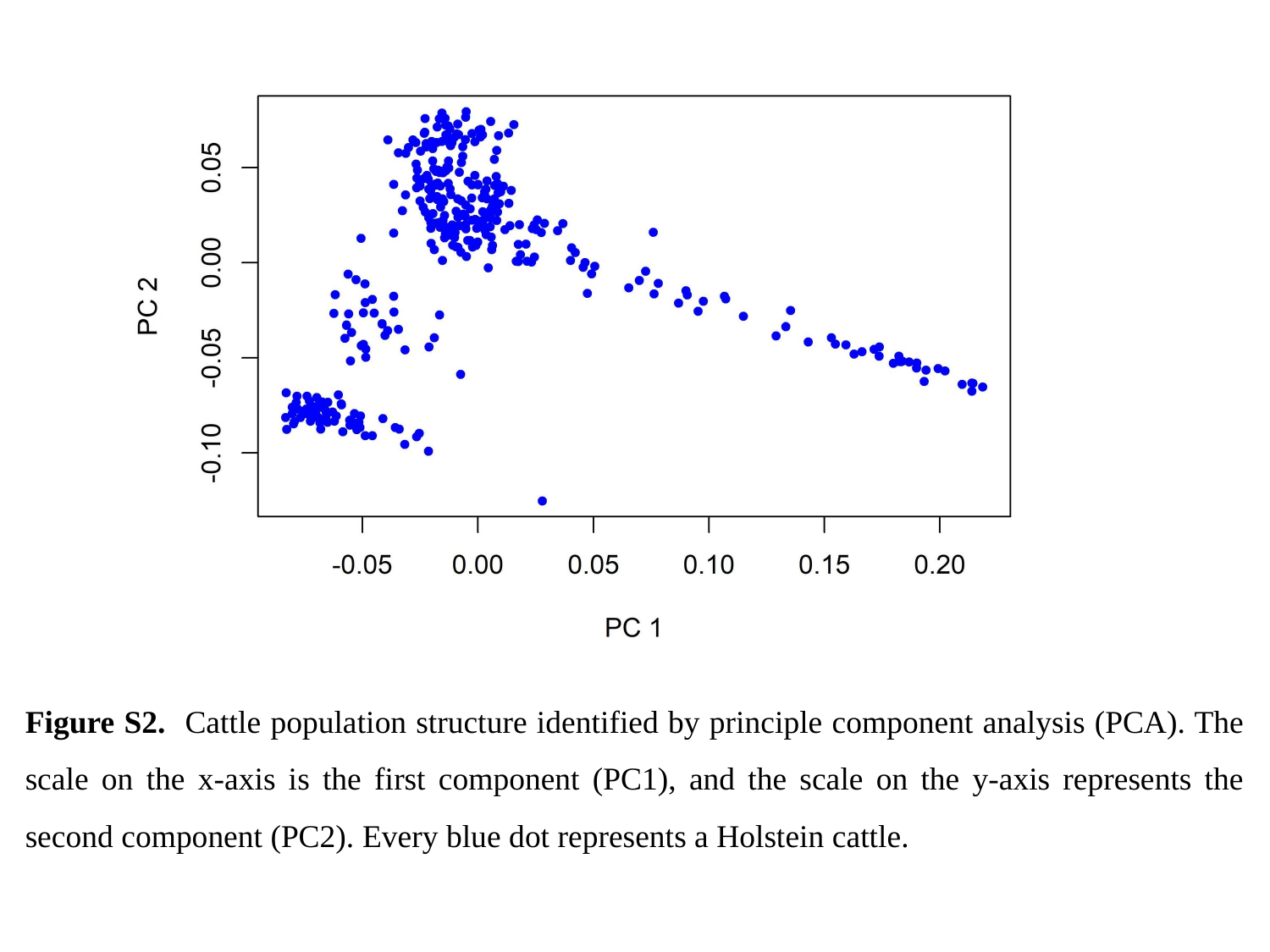

Figure S2. Cattle population structure identified by principle component analysis (PCA). The scale on the x-axis is the first component (PC1), and the scale on the y-axis represents the second component (PC2). Every blue dot represents a Holstein cattle.

## Slide 3
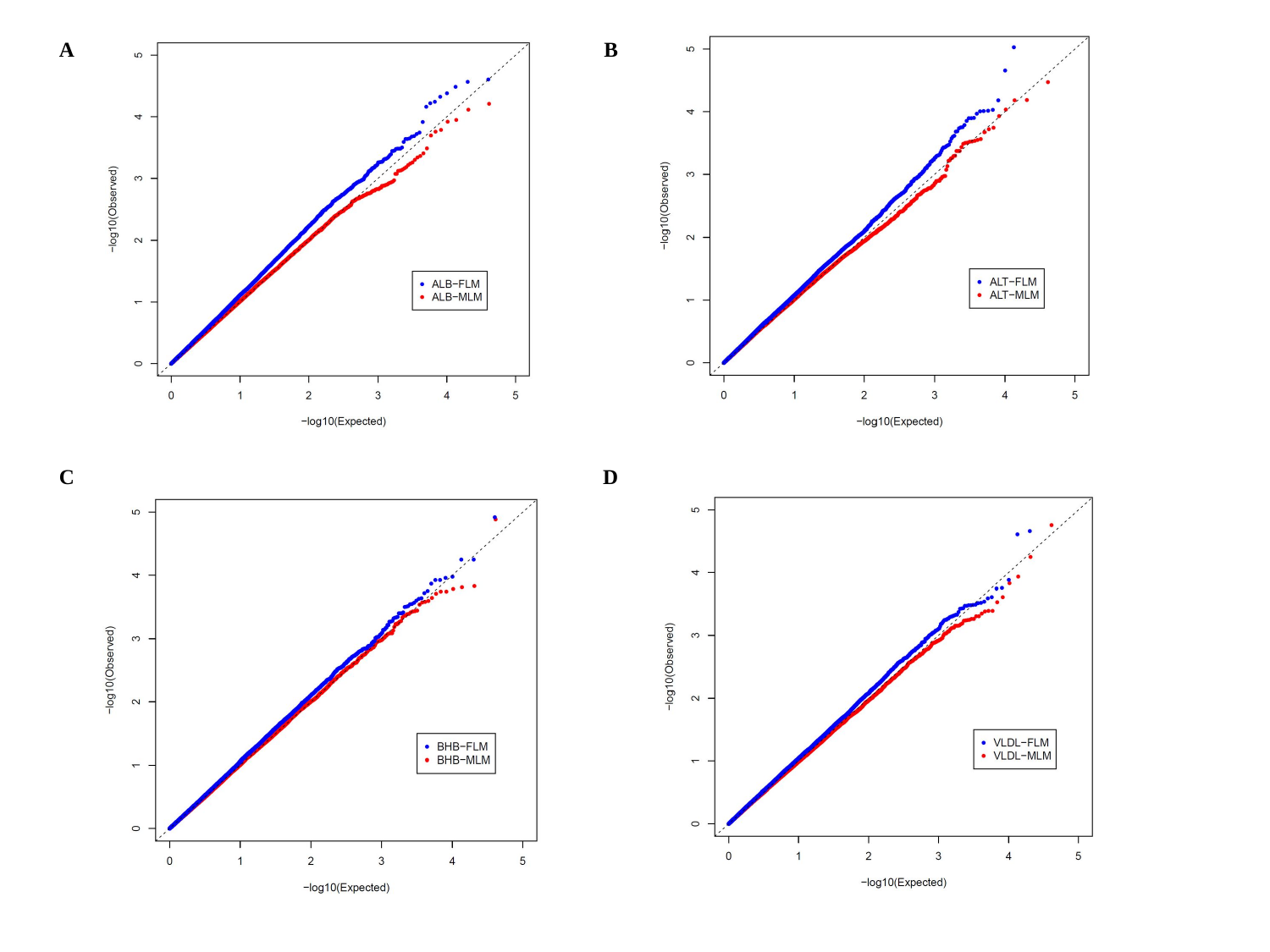

A
B
C
D

## Slide 4
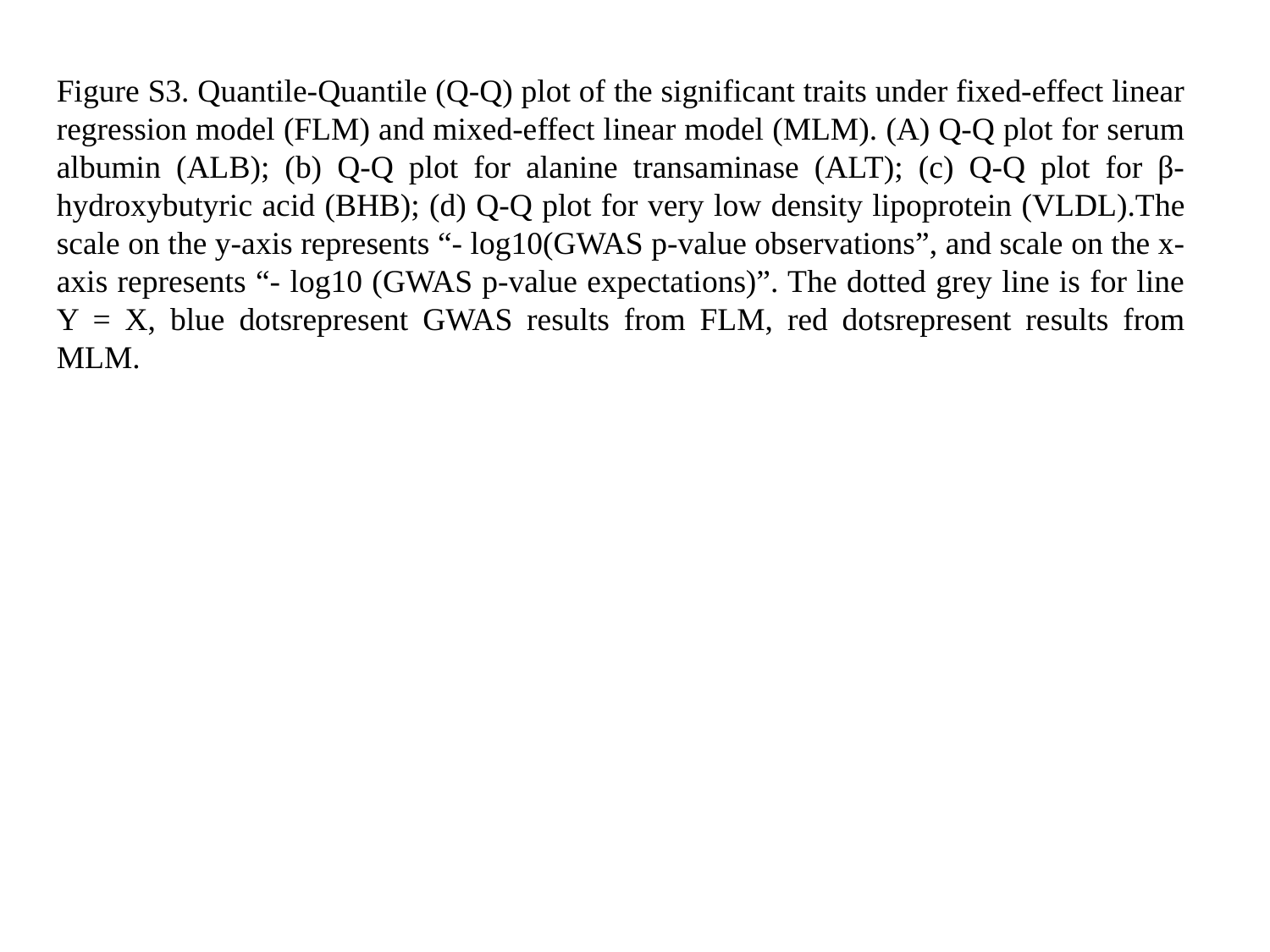

Figure S3. Quantile-Quantile (Q-Q) plot of the significant traits under fixed-effect linear regression model (FLM) and mixed-effect linear model (MLM). (A) Q-Q plot for serum albumin (ALB); (b) Q-Q plot for alanine transaminase (ALT); (c) Q-Q plot for β-hydroxybutyric acid (BHB); (d) Q-Q plot for very low density lipoprotein (VLDL).The scale on the y-axis represents “- log10(GWAS p-value observations”, and scale on the x-axis represents “- log10 (GWAS p-value expectations)”. The dotted grey line is for line Y = X, blue dotsrepresent GWAS results from FLM, red dotsrepresent results from MLM.
